# Trans-catheter aortic valve replacement program in a community hospital – Comparison with US national data

**DOI:** 10.1371/journal.pone.0204766

**Published:** 2018-09-27

**Authors:** Dan Loberman, Shahzad Shaefi, Rephael Mohr, Phillip Dombrowski, Richard B. Zelman, Yifan Zheng, Paul A. Pirundini, Tomer Ziv-Baran

**Affiliations:** 1 Division of Cardiac Surgery, Brigham & Women’s Hospital, Harvard Medical School, Boston, Massachusetts, United States of America; 2 The Heart Center, Cape Cod Hospital, Hyannis, Massachusetts, United States of America; 3 Department of Anesthesia, Critical Care and Pain Medicine, Beth Israel Deaconess Medical Center, Harvard Medical School, Boston, Massachusetts, United States of America; 4 Sackler Faculty of Medicine, Tel Aviv University, Tel Aviv, Israel; 5 School of Public Health, Sackler Faculty of Medicine, Tel Aviv University, Tel Aviv, Israel; University of Mississippi Medical Center, UNITED STATES

## Abstract

Symptomatic aortic stenosis remains a surgical disease, with aortic valve replacement resulting in symptom reduction and improvement in survival. For patients who are deemed a higher surgical risk, Transcatheter aortic-valve replacement (TAVR) is a viable, less invasive and increasingly common alternative. The study compares early outcomes in patients treated within one year of the commencement of TAVR program in a community hospital against outcomes of TAVR patients from nationwide reported data (Society of Thoracic Surgeons/ American College of Cardiology TVT registry). Preoperative characteristics and standardized procedural outcomes of all patients who underwent TAVR in Cape Cod Hospital between June 2015 and May 2016 (n = 62, CCH group) were compared using standardized data format to those of TAVR patients operated during the same time period in other centers within the United States participating in the STS/ACC TVT Registry (n = 24,497, USA group). Most preoperative patient characteristics were similar between groups. However, CCH patients were older (age≥80 years: 77.4% versus 64.3%, p = 0.032) and more likely to be non-elective cases (37.1% versus 9.7%, p<0.001). All 62 TAVR procedures in CCH were performed in the catheterization laboratory unlike most (89.7%) of the procedures in the USA group that were performed in hybrid rooms. A larger proportion of patients in the USA registry underwent TAVR under general anesthesia (78.2% vs.37.1%, P<0.001). Early aortic valve re- intervention rate was 0/62 (0%) in the CCH group VS. 74/ 24,497 (0.3%) in the USA group. In hospital mortality, which was defined as death of any cause during thirty days from date of operation, (CCH: 0% vs. USA: 2.5%, p = 0.410) and occurrence of early adverse events (including postoperative para-valvular leaks, conduction defects requiring pacemakers, neurologic and renal complications) were similar in the two groups. The study concludes that with specific team training and co-ordination, and with active support of experienced personnel, high risk patients with severe aortic valve stenosis can be managed safely with a TAVR procedure in a community hospital.

## Introduction

Surgical replacement of the aortic valve has been shown to reduce symptoms and improve survival in patients with aortic stenosis [1, 2]. However, in clinical practice, at least 30% of patients with severe aortic stenosis do not undergo open heart surgery for replacement of the aortic valve, owing to relative contraindications such as advanced age, left ventricular dysfunction, and/ or the presence of multiple coexisting conditions [1, 3, 4]. Increasingly for these patients, who are deemed as high surgical risk [5, 6], Trans catheter aortic-valve replacement (TAVR) is rapidly becoming a more viable, less invasive and less morbid alternative [7–9]. Still an immature technology, TAVR was approved by the US Food and Drug Administration (FDA) as recently as late 2011 for the treatment of patients with severe symptomatic aortic stenosis who are judged too ill or frail for the traditional surgical aortic valve replacement (SAVR) [10]. Lately, FDA approval has additionally been granted for a wider population of lower risk patients, further expanding the scope of this new technology. The procedure has rapidly gained patient’s and doctor’s acceptance, particularly for the octogenarian and nonagenarian patient populations. Aortic valve procedures have risen more than 60% since 2012, concomitantly with the number of centers performing TAVR procedures, allowing approximately 24000 TAVRs to be performed during 2016 according to the national TAVR registry published by the STS and ACC associations [11].

With more hospitals joining the TAVR providing community, it is evident that some variety exits with regard to structure and systems of care. Characteristics such as hospital volume, teaching status and staffing patterns are known to affect medical and surgical outcomes [12]. Several studies had discussed procedures outcomes in low-volume hospital versus high-volume hospitals in cardiac and non-cardiac surgery and demonstrated that major surgery can be performed safely at community hospital [12–15]. Some of these studies had shown better outcomes in patients undergoing their procedures in a high volume hospital [14–15]. It is as yet unclear whether the volume-outcome relationship has persisted in TAVR.

Outcome of Surgical Aortic Valve Replacement (SAVR) may be related to hospital volume [12]. In contrast, TAVR may be performed in the cardiac catheterization laboratory rendering it more suitable for community hospitals with active catheterization laboratory.

The purpose of this study is to compare characteristics and early outcomes of patients undergoing TAVR in a community hospital, within the first year of initiation of a structural heart disease program, to those of TAVR patients operated during the same time period captured by the STS/ACC TVT registry (USA group) [16].

## Patients and methods

### Study design

This study summarized parameters which were obtained from the STS/ACC TVT Registry [16], making it an ecological study [17]. The study includes all patients reported in the registry who underwent TAVR between June 2015 and May 2016. The study compares characteristics and early outcomes of patients who underwent the procedure in Cape-Cod hospital, a community hospital, to those of patients who underwent the procedure in other all other hospitals in the registry.

The study was approved by the Institutional Review Board (IRB) of Cape Cod Hospital and informed consent was waived (Clinical registry: researchregistry4325).

### Setting

Cape Cod Hospital (CCH) is a 259-bed acute care community hospital located in Hyannis, Massachusetts. CCH has a 15 bed cardiac intensive care unit that serves pre and post-operative cardiac surgery and interventional cardiology patients. Approximately 1800 interventional cardiology cases are done each year, with more than 700 percutaneous interventions (PCI). Cardiac surgery volume is steady over study period with 230 pump cases per year. Since its inception, the TAVR program setup includes a streamlined process of patient evaluation by our valve clinic team, which includes a TAVR program coordinator, two cardiac surgeons, and a cardiologist, and then a scheduled procedure day, at the beginning of which a pre procedural briefing is conducted. During briefing, patient’s clinical and imaging data is presented before the all personnel who take part in the procedure: cath lab and operating room technicians, perfusionists, nurses, surgeons, cardiologist and interventional radiologist. The procedure itself is led by an interventional cardiologist, assisted by either a surgeon, another cardiologist or an interventional radiologist. A surgical backup for an emergency is available at all times. CCH TAVR team includes 3 interventional cardiologists, 2 cardiac surgeons, and 2 cardiac anesthesiologists who provide echo guidance. The TAVR program was started in Cape Cod Hospital in June 2015.

### Participents

Sixty two patients with aortic stenosis underwent TAVR in CCH (CCH group) during the study period. The consecutive population constituted of all sequential TAVR procedures performed in CCH during this time period.

We compared patient characteristics and procedure outcomes of these CCH TAVR patients to those of 24,497 TAVR patients operated during the same time period in other USA centers participating in the STS/ACC TVT Registry (USA group).

### Variables and data source

Summarized baseline patient characteristics (demographic data, co-morbidities, aortic valve description, heart status, and recent heart events), procedure characteristics (procedure location, concurrent PCI, valve-in-valve procedure, type of anesthesia, procedure duration, fluoro-time and other factors presenting Tables 1, 2 and 3 and in-hospital outcomes (cardiac complications, neurologic complications, renal complications, bleeding, major vascular complication, device complication and discharge parameters such as length of stay) were collected from the TVT registry according to The Society of Thoracic Surgeons Adult Cardiac Surgery Database Data Collection Form Version 2.81 (April 23 2015) [6]. Follow-up information was obtained by accessing data from the STS/ACC TVT Registry 2015 and 2016 annual reports [11].

**Table 1:** Comparison of demographic data and co-morbidities of patients underwent the procedure in Cape Cod Hospital (CCH group) versus in other hospitals (USA group).

Factor	Cape Cod Hospital	Other Hospitals	p
	(N = 62)	(N = 24,497)	
Age ≥80	48 (77.4%)	15,758 (64.3%)	0.032
Male	40 (64.5%)	12,939 (52.8%)	0.065
Race			
White	60 (98.4%)	23,046 (94.4%)	0.191
Black	0 (0.0%)	929 (3.8%)	
Other	2 (3.2%)	522 (2.1%)	
Medicare	51 (82.3%)	21,617 (88.4%)	0.130
BMI ≥ 35	13 (21.0%)	3,506 (14.3%)	0.135
NYHA Class ≥ III	50 (80.6%)	19,830 (81.3%)	0.952
Five meter walk > = 6 sec.	34 (58.6%)	14,207 (66.9%)	0.196
Home Oxygen	2 (3.2%)	2,752 (11.2%)	0.046
CRF (Cr >2.0 mg/dl, ex. dialysis)	0 (0.0%)	1,213 (5.2%)	0.077
Dialysis	1 (1.6%)	1,041 (4.3%)	0.524
Immunocompromised	6 (9.7%)	2,543 (10.4%)	0.852
Prior Stroke	3 (4.8%)	2,994 (12.2%)	0.076
Carotid Stenosis	17 (30.4%)	5,312 (26.4%)	0.501
Prior CEA	5 (8.9%)	1,741 (8.6%)	0.811
Procedure Status			
Elective	39 (62.9%)	22,151 (90.4%)	<0.001
Urgent	21 (33.9%)	2,290 (9.3%)	
Emergency	2 (3.2%)	72 (0.3%)	

BMI- body mass index; NYHA- New York Heart Association; CRF- Chronic Renal Failure; CEA- Carotid Endarterectomy.

**Table 2:** Comparison of aortic valve description, heart status, and recent heart events of patients underwent the procedure in Cape Cod Hospital (CCH group) versus in other hospitals (USA group).

Factor	Cape Cod Hospital	Other Hospitals	p
	(N = 62)	(N = 24,497)	
Degenerative disease	58 (93.5%)	23,212 (94.8%)	0.658
Prior Valve Proc.	7 (11.3%)	3,414 (13.9%)	0.548
Prior SAVR	3 (4.8%)	1,425 (5.8%)	>0.999
AV repair	2 (3.2%)	210 (0.9%)	0.100
AV Balloon Valvuloplasty	4 (6.5%)	1,878 (7.7%)	>0.999
TAVR	0 (0.0%)	123 (0.5%)	>0.999
Other Trans-catheter Int.	0 (0.0%)	45 (0.2%)	>0.999
Prior Non-aortic Valve Proc.	2 (3.2%)	630 (2.6%)	0.681
**Heart status**			
Atrial Fibrillation	28 (45.2%)	9,982 (40.8%)	0.484
Endocarditis	1 (1.6%)	245 (1.0%)	0.459
Permanent Pacemaker	6 (9.7%)	3,787 (15.5%)	0.208
Previous ICD	2 (3.2%)	1,053 (4.3%)	0.588
Left Main Disease (≥50%)	7 (11.9%)	2,384 (9.8%)	0.594
Proximal LAD (≥70%)	14 (23.7%)	4,676 (19.2%)	0.379
Previous Cardiac Surgery	11 (17.7%)	7,090 (28.9%)	0.052
Prior CABG	11 (17.7%)	6,364 (26.0%)	0.140
Porcelain Aorta	1 (1.6%)	1,230 (5.0%)	0.375
Conduction Defect	6 (10.2%)	9,105 (37.4%)	<0.001
Recent heart events			
Prior MI	17 (27.4%)	6,011 (24.6%)	0.606
MI 30 days prior to the procedure	10 (16.1%)	649 (2.7%)	<0.001
Heart Failure w/in 2 weeks	24 (38.7%)	19,509 (79.6%)	<0.001
Cardiogenic Shock w/in 24 hrs	2 (3.2%)	166 (0.7%)	0.070
Cardiac Procedure w/in 30 days	3 (4.9%)	2,296 (9.4%)	0.230
Pre-op. Anticoagulants	14 (22.6%)	7,826 (32.0%)	0.111
Pre-op. Inotropes	2 (3.2%)	669 (2.8%)	0.693
**Aortic Annulus Sizing Method**			
TTE	1 (1.6%)	3,076 (13.7%)	0.006
TEE	2 (3.3%)	3,310 (14.8%)	0.011
CTA	57 (93.4%)	15,981 (71.3%)	<0.001
**ECHO findings**			
Ejection Fraction			
Mild Dysfunction (≥40%)	49 (81.7%)	20,416 (83.8%)	0.697
Mod. Dysfunction (30–39%)	7 (11.7%)	2,110 (8.7%)	
Severe Dysfunction (<30%)	4 (6.7%)	1,848 (7.6%)	
Aortic Regurgitation			
None-Mild	51 (83.6%)	19,197 (78.7%)	0.580
Moderate	7 (11.5%)	4,002 (16.4%)	
Severe	3 (4.9%)	1,194 (4.9%)	
Annulus Calcification	59 (96.7%)	19,131 (79.3%)	0.001
Aortic Stenosis			
Mean gradient <20 mmHg	0 (0.0%)	811 (3.4%)	0.186
Mean gradient 20–40 mmHg	21 (35.0%)	9,717 (40.6%)	
Mean gradient >40 mmHg	39 (65.0%)	13,395 (56.0%)	
Mitral Regurgitation			
None-Mild	40 (70.2%)	13,397 (65.3%)	0.389
Moderate	12 (21.1%)	5,834 (28.4%)	
Moderate/severe to Severe	5 (8.8%)	1,278 (6.2%)	
Mitral Stenosis	8 (14.5%)	2,785 (13.8%)	0.872
Tricuspid Regurgitation			
None-Mild	47 (77.0%)	18,394 (75.6%)	0.940
Moderate	11 (18.0%)	4,817 (19.8%)	
Severe	3 (4.9%)	1,128 (4.6%)	

SAVR- Surgical Aortic Valve Replacement; AV- Aortic Valve; TAVR- Transcatheter Aortic Valve Replacement; Int.- Intervention; Proc.- Procedure. ICD- Implantable Cardioverter Defibrillator; LAD- Left anterior descending artery; CABG- Coronary artery bypass grafting; MI- Myocardial infarction; W/in- Within; Pre-op- Preoperative. TTE- Trans Thoracic Echo; TEE- Trans Esophageal Echo; CTA- Computed Tomography Angiography; Mod- Moderate; Reg- Regurgitation.

**Table 3:** Comparison of procedures' parameters performed in patients underwent the procedure in Cape Cod Hospital (CCH group) versus in other hospitals (USA group).

Factor	Cape Cod Hospital	Other Hospitals	p
	(N = 62)	(N = 24,497)	
Procedure Location			
Hybrid or Suite	0 (0.0%)	15,209 (62.1%)	<0.001
Hybrid Cath. Lab	0 (0.0%)	6,759 (27.6%)	
Cath Lab.	62 (100%)	2,513 (10.3%)	
Concurrent PCI	5 (8.1%)	459 (1.9%)	0.006
Procedure Indication			
Primary AS	58 (93.5%)	22,861 (93.3%)	<0.001
Primary AI	2 (3.2%)	186 (0.8%)	
Mixed AS/AI	1 (1.6%)	616 (2.5%)	
Failed Bio-prosthetic	1 (1.6%)	848 (3.5%)	
Valve-in-Valve Procedure	4 (6.5%)	1,840 (7.5%)	>0.999
Type of Anesthesia			
Moderate sedation	39 (62.9%)	5,184 (21.2%)	<0.001
General anesthesia	23 (37.1%)	19,140 (78.2%)	
Combination+Epidural	0 (0.0%)	148 (0.6%)	
Patients with>1 valve	2 (3.2%)	504 (2.1%)	0.375
Procedure Duration (min)			
Median (IQR)	100 (78–124)	103 (78–140)	0.670
Fluoro-Time (minutes)			
Median (IQR)	25.5 (21–32)	18.6 (14–25)	<0.001
Contrast Volume			
Median (IQR)	150 (126–180)	100 (65–150)	<0.001
Femoral Access	62 (100%)	21,786 (89.1%)	0.006
Assessed risk for SAVR			
Extreme	6 (9.7%)	5,749 (23.5%)	<0.001
High Risk	33 (53.2%)	17,430 (71.2%)	
Intermediate risk	10 (16.1%)	1,054 (4.3%)	
Low risk	13 (21%)	252 (1%)	
Post-procedure Inotropes	14 (22.6%)	10,281 (42%)	0.002
Post-procedure IABP	1 (1.6%)	61 (0.2%)	0.145
Assist with CPB	1 (1.6%)	309 (1.3%)	0.550
Conversion OH surgery	1 (1.6%)	180 (0.7%)	0.368

OR, Operating Room; Cath. Lab., Catheterization Laboratory; PCI, Per-Cutaneous Intervention; AS, Aortic Stenosis; AI, Aortic Insufficiency; IQR, Inter Quartile Range; IABP, Intra Aortic Balloon; CPB, Cardio Pulmonary Bypass; min, minutes; ml, milliliter; SAVR, Surgical Aortic Valve Replacement; OH, Open Heart.

Data was summarized as mean, median, interquartile range, range, number and percentage by the STS from the STS/ACC TVT Registry and the summarized data was compared between the two studied populations.

### Bias

In order to avoid potential bias we used only retrieved summarized data that was based on reports to the STS using a uniform data collection form.

### Statistical analysis

Categorical variables were expressed as number and percentages and continuous variables were described using mean (standard deviation, SD), and median (interquartile range, IQR).Standard deviation was estimated using the IQR and sample size [18]. Categorical variables were compared using Chi-square test or Fisher's exact test and continuous variables were compared using the independent samples t-test. A two-tailed p<0.05 was considered statistically significant. Analyses were performed with OpenEpi [19] and SPSS (IBM Corp. Released 2016. IBM SPSS Statistics for Windows, Version 24.0. Armonk, NY: IBM Corp.).

### Results

**Fig 1:**
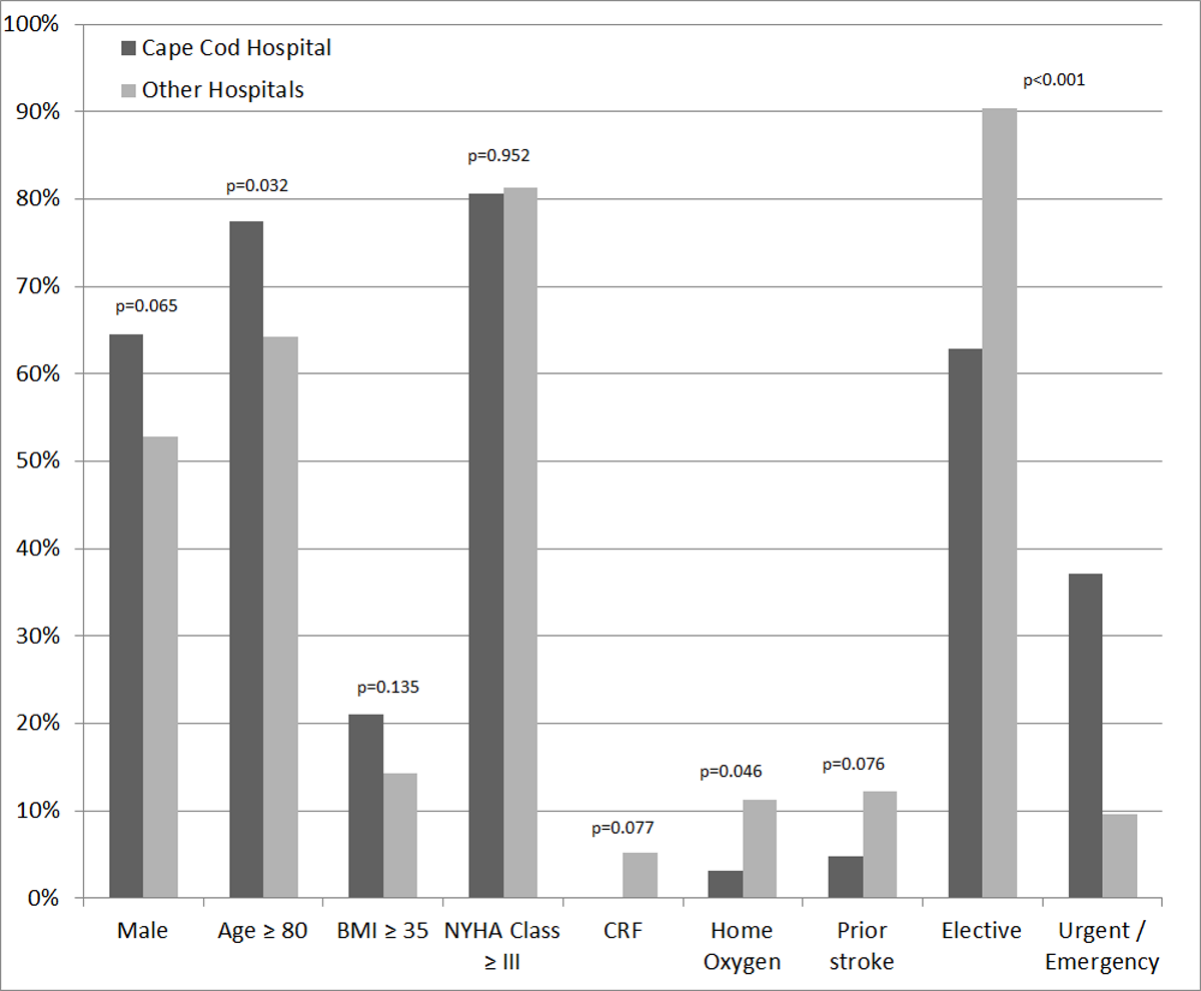
Comparison of patients’ demographic and co-morbidity between Cape Cod and other hospitals.

The vast majority of preoperative patient demographic characteristics and co-morbidities were similar between groups. However patients in CCH were older, 77.4% of the CCH patients were 80 or older compared to 64.3% of the TVT patients (p = 0.032). Patients in CCH were more likely to be non-elective cases (37.1% versus 9.6%, p<0.001) and more likely to have private health insurance (90.3% versus 63.7%, p<0.001). On the other hand more patients in the USA group were on home oxygen therapy before the procedure. Patients’ characteristics and pre-procedure and heart status are presented (Tables 1 and 2 and Fig 1). Occurrences of pre-procedure degenerative aortic stenosis, prior SAVR or aortic valve repair and prior balloon valvuloplasty or TAVR were not significantly different between groups (Table 2).

The prevalence of previous cardiac surgery (28.9% vs. 17.7% p = 0.052) and pre-procedure conduction defect (37.4% vs. 10.2%, p<0.001) was higher among patients in the national TAVR registry. In contrast, prior MI (16.1% vs.2.7%, p<0.001) and heart failure (79.6% vs. 38.7%, p<0.001) were more prevalent among CCH patients characteristics (Table 2).

Computed Tomographic Angiography (CTA) was more commonly used in CCH as means for aortic annular sizing (93.4% vs. 71.3%, p<0.001). Trans-esophageal echocardiography (TEE) was more common among the USA group patients (14.3% vs. 3.3%, p = 0.011) (Table 2).

All 62 TAVR procedures in CCH were performed in the catheterization laboratory unlike most (89.7%) of the TAVR in the USA group that were performed in hybrid operating rooms or hybrid laboratories. A larger proportion of TAVR patients in the USA group underwent the procedure under general anesthesia (78.2% vs. 37.1%, p<0.001)) and more patients in this registry were extreme or higher risk for SAVR (p<0.001). Procedural characteristics are presented (Table 3).

**Fig 2:**
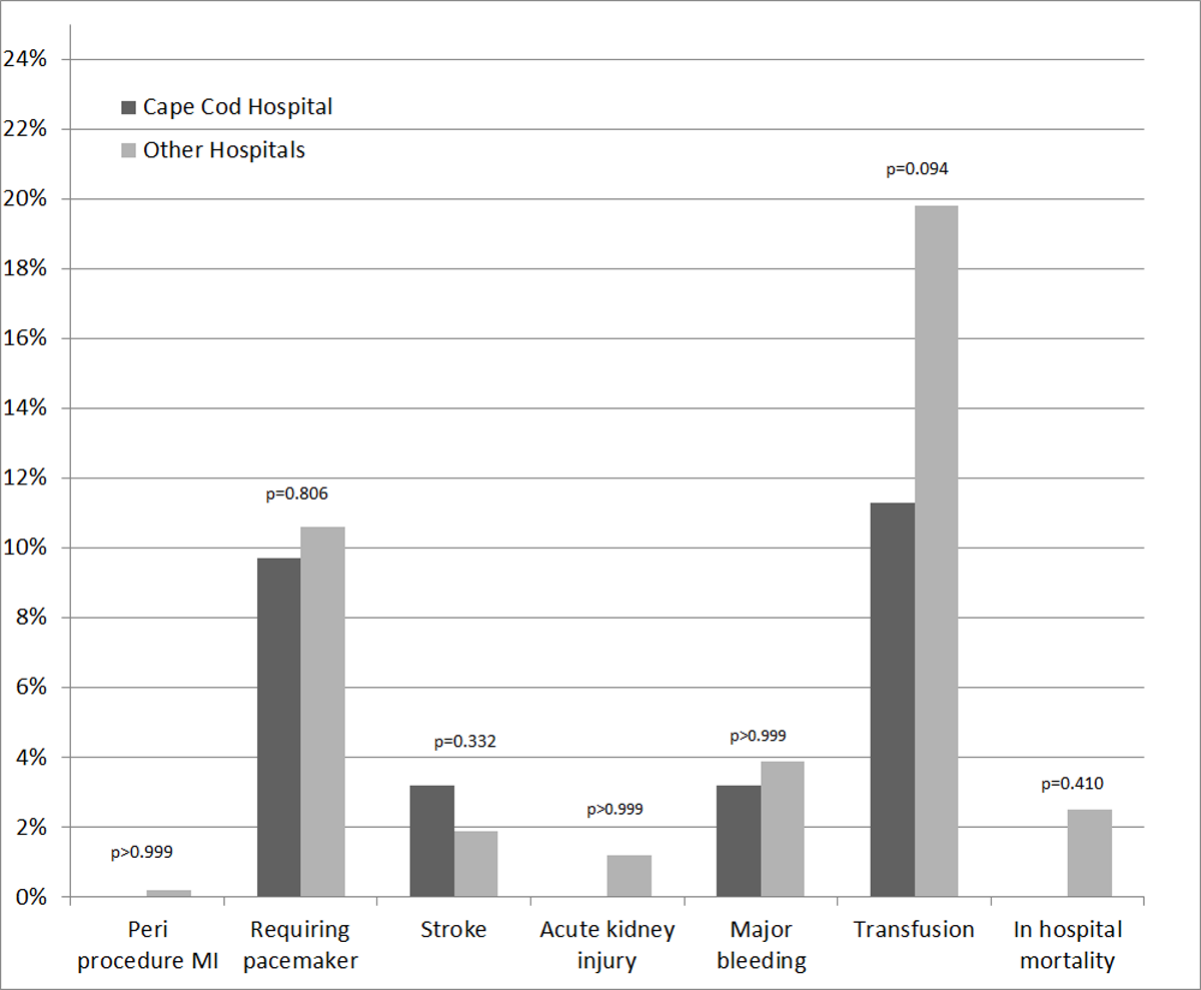
Comparison of early outcomes between Cape Cod and other hospitals.

The rate of early adverse events (including all cardiac, neurologic and renal complications) as well as the immediate post-procedure echo findings was similar in the two groups (Table 4 and Figs 1 and 2). In hospital mortality was also similar in the two groups (Table 4). Seventy four out of the 24497 patients in the USA group (0.3%) had to go for an emergent/salvage aortic valve re-intervention procedure, while none of the patients from the CCH group needed such intervention.

**Table 4:** Comparison of early outcome, post–procedure echo findings, and length of stay of patients underwent the procedure in Cape Cod Hospital (CCH group) versus in other hospitals (USA group).

Factor	Cape Cod Hospital	Other Hospitals	p
	(N = 62)	(N = 24,497)	
**Any complication**	16 (25.8%)	6,813 (27.8%)	0.725
**Cardiac complications**			
Procedure related	0 (0.0%)	386 (1.6%)	>0.999
Peri-procedure MI	0 (0.0%)	54 (0.2%)	>0.999
Endocarditis	0 (0.0%)	3 (0.0%)	>0.999
Conductive disturbance	8 (12.9%)	2,731 (11.1%)	0.661
Requiring pacemaker	6 (9.7%)	2,606 (10.6%)	0.806
Requiring ICD	2 (3.2%)	131 (0.5%)	0.045
Cardiac arrest	1 (1.6%)	747 (3.0%)	>0.999
Atrial fibrillation	0 (0.0%)	847 (3.5%)	0.279
Annular dissection	0 (0.0%)	49 (0.2%)	>0.999
Aortic dissection	0 (0.0%)	35 (0.1%)	>0.999
Perforation ± Tamponade	0 (0.0%)	277 (1.1%)	>0.999
**Neurologic complications**			
TIA	0 (0.0%)	54 (0.2%)	>0.999
Stroke	2 (3.2%)	467 (1.9%)	0.332
**Renal complications**			
Acute kidney injury (stage 3)	0 (0.0%)	282 (1.2%)	>0.999
Dialysis	0 (0.0%)	219 (0.9%)	>0.999
**Bleeding**			
Major Bleeding	2 (3.2%)	963 (3.9%)	>0.999
Life Threatening Bleeding	0 (0.0%)	821 (3.4%)	0.274
Hematoma at access site	1 (1.6%)	410 (1.7%)	>0.999
Retroperitoneal hematoma	0 (0.0%)	119 (0.5%)	>0.999
GI bleed	0 (0.0%)	173 (0.7%)	>0.999
GU bleed	0 (0.0%)	89 (0.4%)	>0.999
Transfusion (RBC/whole blood)	7 (11.3%)	4,846 (19.8%)	0.094
**Major vascular complication**	0 (0.0%)	316 (1.3%)	>0.999
**Vascular Surgery / Intervention**	1 (1.6%)	824 (3.4%)	0.725
**Device complication**	2 (3.2%)	337 (1.4%)	>0.999
**PCI**	2 (3.2%)	74 (0.3%)	>0.999
**Aortic Stenosis**			
Mean gradient <20 mmHg	59 (95.2%)	19589 (94.2%)	>0.999
Mean gradient 20–40 mmHg	3 (4.8%)	1113 (5.4%)	
Mean gradient >40 mmHg	0 (0.0%)	89 (0.4%)	
Aortic Regurgitation			
None-Trace	53 (85.5%)	15440 (73.3%)	0.148
Mild	9 (14.5%)	4783 (22.7%)	
Moderate	0 (0.0%)	778 (3.7%)	
Severe	0 (0.0%)	54 (0.3%)	
Para-valvar AR*			
None	4 (26.7%)	3512 (37.2%)	0.527
Mild	11 (73.3%)	5203 (55.2%)	
Moderate	0 (0.0%)	672 (7.1%)	
Severe	0 (0.0%)	44 (0.5%)	
**Discharge**			
Discharge Status			
Alive	62 (100%)	23884 (97.5%)	0.410
Deceased	0 (0.0%)	613 (2.5%)	
Discharge Location			
Home	47 (75.8%)	18441 (77.2%)	0.232
Extended care / Rehab.	8 (12.9%)	4042 (16.9%)	
Other hospital	0 (0.0%)	129 (0.5%)	
Nursing home	7 (11.3%)	1137 (4.8%)	
Other	0 (0.0%)	128 (0.5%)	
Discharge Medications			
Dabigatran	0 (0.0%)	333 (1.4%)	>0.999
Factor Xa Inhibitor	0 (0.0%)	1901 (8%)	0.015
Warfarin	26 (41.9%)	5547 (23.2%)	0.001
ACE Inhibitor	27 (43.5%)	7658 (32.1%)	0.053
ARB	7 (11.3%)	4275 (17.9%)	0.175
Beta Blockers	36 (58.1%)	17170 (71.9%)	0.015
Anti-arrhythmic	9 (14.5%)	3638 (15.2%)	0.875
**Length of stay (LOS)**			
Total LOS (days), Median (IQR)	3 (2–8)	4 (3–7)	0.731
ICU stay (hours),Median (IQR)	28.5 (24–47)	27.5 (22–49)	0.001
Post-procedure LOS (days)			
Median (IQR)	2 (2–3)	3 (2–5)	<0.001
Post-proc LOS <6 days	56 (90.3%)	18413 (75.2%)	0.006
Post-proc LOS >14 days	0 (0.0%)	836 (3.4%)	0.277

MI, Myocardial Infarction; ICD, Implantable Cardioverter Defibrillator; TIA, Transient Ischemic Attack; GI, Gastro-intestinal; GU, Genito-urinary; RBC, Red Blood Cells; Interv, Intervention; PCI, Percutaneous AR- Aortic Regurgitation;

* Number evaluated for para-valvar AR; LOS- Length Of Stay; ACE- Angiotensin Converting Enzyme; IQR- Inter Quartile Range; ICU- Intensive Care Unit; Rehab- Rehabilitation.

## Discussion

The selection of our TAVR patients described reflects typical contemporary referral patterns for this minimal invasive procedure. These are mostly elderly patients with severe aortic stenosis who are judged by a Heart Team, including two cardiac surgeons, to be at high risk for SAVR (i.e., predicted risk of surgical mortality >8% at 30 days), based on the Society of Thoracic Surgeons (STS) risk score [20]. A portion of our patient cohort had lower STS scores. However, after being reviewed by our valve clinic physicians, other parameters that are not included in the STS score were given weight. For instance, a 52 year old female, robust and active, who was status post sternotomy for Goiter removal, and then had heavy radiation to the chest d/t a different malignancy—has a very low calculated STS risk score, but a very high operative risk. All patients deemed adequate for TAVR had one or more of these “other parameters” that made the team favor TAVR over SAVR.

Our study compared one year (June 2015- May 2016) results and patients characteristics of TAVR patients treated in a community hospital (CCH) to those of 24,497 TAVR patients operated during the same time period in USA centers participating in the STS/ACC TVT Registry.

There was higher prevalence of non–elective cases in the CCH group. This is probably the reason for higher proportion of post- acute MI, older patients and patients with new heart failure in this group. There was no statistical difference in procedure time between the two groups (Table 3). However, the significantly higher volume of contrast material in CCH compare to the USA group may be related to lack of experience in CCH.

The main finding in this report is the similar outcome (including in hospital mortality and early adverse events) of patients operated in community hospital to that of patients operated in hospital in the STS/ACC TVT registry. Early outcome of CCH TAVR patients was similar to the outcome in the TVT registry despite the fact that there were more emergent and urgent patients among the CCH patients and that their proportion of patients above the age of 80 years was significantly higher. Outcomes such as decreased length of stay may be related to the higher use of moderate sedation in CCH compare to the USA group. Although not previously trained in treating structural heart defects, the leading interventional cardiologist at CCH is a seasoned interventionalist, who has been steadily performing roughly 700 PCI cases each year for the last 10 years. Prior to embarking on the TAVR program, our interventional cardiologist used the available training programs for specific products, took use of available case observation programs, and used a specialized proctor for the first few cases. A fellowship trained cardiac surgeon, an interventional radiologist and a very experienced anesthesiology team were instrumental in safely conducting the first 50 cases, forming the “learning curve”. The operators do not spend time in tertiary centers. This may be related to skills of an experienced cardiologist performing the TAVR in CCH, to the active involvement of an interventional radiologist in TAVR cases which might contribute to reduction in vascular injuries, and specifically to the role of a very experienced cardiac anesthesiology group, which implemented a 5 year experience accumulated while building a similar program in a large referral center beforehand. This resulted in higher rates of partial sedation vs. general anesthesia from a very early stage during the program development, which might have contributed to faster recovery from intensive care related issues, and favorable early outcome.

Improvements in trans-catheter valve systems and devices have led expansion of TAVR procedures from the large academic hospitals to smaller institutions performing SAVR. TAVR, performed in experienced centers, with the use of a lower-profile, 2nd-generation device, was non-inferior to surgery with respect to death from any cause or disabling stroke at 2 years. Their bio-prosthetic-valve gradients were lower and the valve areas were greater, as compared with surgical valves [21]. Similar findings were reported recently with the third generation TAVR devices (Edward Sapian 3) in recently published studies describing outcome in intermediate -risk (STS score 4–8) aortic valve patients [22–24]. Other devices were not used in our patient cohort.

In CCH the TAVR program was started in 2015. All cases were done with balloon expandable valves. Sapien XT was used in the first 8 cases, after which the Sapien 3 was used in essentially all cases. The use of the Edwards Sapien XT (2^nd^ generation) and Sapien 3(3^rd^ generation) with accompanying team [25] and the use of multislice computed tomography to assess aortic annulus dimensions for appropriate valve sizing [26] can explain the relatively short learning curve and the satisfactory early outcome achieved in intermediate as well as high risk TAVR patients.

In the early TAVR studies coronary artery disease (CAD) was an indication for SAVR and contra-indication for TAVR, however, in CCH CAD was not a contra-indication for TAVR. More than 35% of the TAVR patients in CCH had significant CAD (≥ 2 vessel disease), 17.7% had prior CABG and 3.2% underwent Concomitant PCI due to critical coronary lesion anatomy, never due to ongoing/ concomitant ischemia.

Outcomes of TAVR procedures performed in large referral centers have been described previously [27]. To the best of our knowledge the outcomes of TAVR in community setting were not reported yet. Occurrences of adverse events among CCH patients were not significantly different than the rate of these complications in the TVT registry.

Our results are not in accordance with results of several previously published reports that had shown that hospital and surgeon procedure volumes are important determinants of patient’s outcomes [13–15]. Those studies had shown better outcomes in patients undergoing their procedures, including surgical aortic valve replacement (SAVR) procedures, in a high volume hospital [14, 15].

The above studies include mostly SAVR patients operated before the TAVR era. Thus, they do not reflect the effect of TAVR introduction on reduction of aortic stenosis patients referred to surgery [6, 16], and cannot therefore reflect the effect of hospital-volume on outcome today.

In addition, conclusions based on the above studies cannot be implemented to TAVR studies due to the fact that TAVR is a smaller procedure that may be performed in the cardiac catheterization laboratory or hybrid operating rooms without extra-corporeal circulation. The number of TAVR procedures performed is mainly related to the size of the referral population which is not always related to the hospital volume.

Due to the relatively short learning curve, our results in CCH may serve as a model for successful initiation of TAVR programs in other community hospitals. In the current healthcare market, there is a tendency to maintain costs and to continue to provide state-of-the-art healthcare.

The proximity of the community hospitals to patients’ homes can both reduce expenses and increase satisfaction for patients and their relatives before and after the procedure, compared to referral and care in large-volume hospitals. With the growing number of patients treated with TAVR in CCH and the expected increased operator experience, further reduction in post-procedure complications is anticipated, although probably minor at this stage, as the learning curve reaching a plateau.

Our study has several limitations. First, it is important to note this study is an ecological registry based study that focuses on the comparison of groups rather than individual subjects. Individual level data are missing, thus direct assessment of exposure-outcome association is not possible. Second, there was limited number of patients operated during the early period that TAVR program was started in CCH. Third, data on long term outcomes was not available. Fourth, small hospital in the big city would be ideal clinical setting that could get benefit from the result of this study rather than a standalone community hospital in the rural area.

## Conclusion

In conclusion, this report describes the initial experience with TAVR in community hospital.

Comparison with the USA national TVT registry was performed in order evaluate early outcome. This comparison shows that successful adoption of TAVR technology by the teams in a community hospital with similar results is feasible.
